# NR4A1-3 nuclear receptor activity and immune cell dysregulation in rheumatic diseases

**DOI:** 10.3389/fmed.2022.874182

**Published:** 2022-07-22

**Authors:** Evelyn P. Murphy, Daniel Crean

**Affiliations:** ^1^School of Medicine, University of Limerick, Limerick, Ireland; ^2^School of Veterinary Medicine, University College Dublin, Dublin, Ireland

**Keywords:** NR4A nuclear receptors, rheumatic disease, innate immunity, inflammation, immune tolerance, inflammatory resolution

## Abstract

The development and progression of immune-mediated rheumatic disease (IMRD) involves dysfunction of innate and adaptive immune cell populations leading to altered responses including inflammasome activation, dysregulated cytokine networks, increased immune cell numbers and multifaceted cell-cell communication. Several rheumatic diseases are further characterized by the presence of autoantibodies, immune complex mediated complement activation and the deficit of peripheral immune tolerance due to reduced regulatory T-lymphocyte cell function. Ultimately, in rheumatic disease the loss in cellular and tissue homeostasis culminates in the advancement of chronic inflammation. The three members of the NR4A subfamily of nuclear receptors are immediate early genes, and act as potent transcriptional responders to changes in the cellular and tissue microenvironment. Subfamily members are rapidly expressed in diseases characterized by inflammation and function to control the differentiation and activity of innate and adaptive immune cells in a cell-type and cell-context specific manner. Rheumatic disease including rheumatoid-, psoriatic-, osteo-arthritis and systemic sclerosis display altered NR4A1-3 activity in controlling immune cell migration and function, production of paracrine signaling molecules, synovial tissue hyperplasia, and regulating cartilage turn-over *in vivo*. Additionally, NR4A1-3 activities mediate cytokine, prostanoid and growth factor signaling to control angiogenesis, modulate the regulatory functions of mesenchymal stromal cells, alter the activation status of dendritic cells, influence the generation of peripheral myeloid and T-lymphocyte lineages and promote the maintenance of functional regulatory T-cells. Further reports uncover the potential of moderating NR4A 1-3 receptors as therapeutic targets in altering immune tolerance, pathological angiogenesis and controlling inflammation in several models of disease.

## Introduction

Immune-mediated rheumatic diseases (IMRDs) are a diverse group of conditions that primarily affect the joints, bones, muscle, and connective tissues of the body. These diseases include rheumatoid arthritis (RA), psoriatic arthritis (PsA), axial spondyloarthritis (AxSpA) and systemic sclerosis (SSc); all of which are described by sustained innate and adaptive immune responses to normal cells and tissues, leading to chronic inflammation, dysregulated repair mechanisms and progressive disease resulting in articular and/or systemic damage (1–3). Rheumatic disease further includes osteoarthritis (OA) which is also a chronic disease characterized by biomechanical deterioration of joint tissues (4, 5). In this review, we outline how IMRDS display altered cell signaling and activity in controlling innate and adaptive immune cell migration and activity, the production of auto- and paracrine signaling molecules leading to altered tissue hyperplasia and tissue turn-over *in vivo*. We explain recent evidence highlighting the contribution of NR4A1-3 receptor activity during the course of IMRDs, primarily focusing on their cell-specific functional activities during disease progression. Cell-specific events regulated by NR4A1-3 receptor activity include angiogenesis, the activation and tolerogenic status of dendritic cells, the homeostasis and lifespan of neutrophils, the generation and responses of peripheral myeloid and T-lymphocyte lineages, the maintenance of functional regulatory T-cells and the regulatory and invasive functions of resident mesenchymal fibroblast cells. Furthermore, we examine reports that reveal the potential of moderating NR4A 1-3 receptors as therapeutic targets in distinct models of rheumatic disease.

## Cell and cytokine signatures in rheumatic diseases

IMRDs are a heterogeneous group of diseases and display differences in genetic susceptibility loci, immune system activation, environmental risk factors and treatment response patterns (6–8). Recent approaches to further understand the pathogenic mechanisms of IMRDs, combined with molecular analysis of disease response following targeted cytokine therapy, are providing critical molecular characterization and mechanistic knowledge of the shared pathophysiology and differences that exist across IMRDs (1). Increased micro-vessel density is one of the earliest histopathologic findings in disease pathogenesis and angiogenesis is required for immune cell [leukocyte and lymphocyte] migration, promoting the development and maintenance of chronic inflammation (9). Cross-talk between the cellular and the autocrine/paracrine mediators of innate and adaptive immune responses is central to underlying disease processes. A shared effector phase in IMRDs is the activation of bone marrow–derived macrophages, dendritic cells, poly-morphonuclear neutrophils, and stromal fibroblast cells [including fibroblast-like synoviocytes (FLS)] which are resident cells at sites of inflammation; resulting in aberrant intercellular communication, persistent activation associated with the production of increased amounts of pro-inflammatory mediators including tumor necrosis factor (TNF) which is considered a pivotal effector cytokine regulating joint pathogenesis (10). Detailed profiling of cell and cytokine signatures in IMRDs indicate that significant differences exist in genetic features, immune-mediated pathogenesis, and treatment responses to targeted therapies (recently reviewed in 1, 11, 12).

The principal cells, the cellular components and disease mechanisms which govern the inflammatory pathogenesis of these IMRDs, with an emphasis on the interplay between innate and adaptive immunity (1, 11, 12), are summarized below:

Rheumatoid Arthritis (RA) disease is further characterized by transformation of synovial membrane into an aggressive, tumor like structure called pannus which develops on the basis of a breach of immune tolerance involving dendritic cells, activated neutrophils, follicular (Tfh) and peripheral helper (Tph) T cells, granzyme K^+^ T cells and B cells in the lymph nodes and synovial tissue. The dysfunction of regulatory T cells (CD4^+^ Fox3P^+^ Tregs) precedes the loss of self-tolerance and the progression of disease. CD4^+^Th cell express Interleukin-17 family members (IL-17A and F) to synergise and amplify local and systemic TNF modulatory outcome(s). These activities lead to plasma-cell differentiation and autoantibody (AAb) production (RF, anti-CCP), as well as activation of FLS with robust Interleukin-6 (IL-6) and Interleukin-8 (IL-8) production and release. Recent evidence from single-cell sequencing indicates that a major product of RA FLS is IL-6, which in addition to chemokines, coordinates early and sustained vascular changes promoting the influx of immune cells to the inflamed site.

Psoriatic Arthritis (PsA) is characterized by enthesitis/synovitis and is closely associated with skin psoriasis as an important source of systemic cytokine release. Pro-inflammatory mediators are additionally produced by resident FLS cells (IL-6) and cells of the innate immune system such as dendritic cells and macrophages (IL-23) to facilitate the differentiation of Th17 cells and impede regulatory T (Treg) cell activity to drive disease pathogenesis. Furthermore, pro-inflammatory lipids such as prostaglandin E2 (PGE2) are produced in the context of mechano-inflammation in the entheses. Both IL-23 and PGE2 stimulate interleukin-17A [IL-17] production by Th17 cells (comprising CD4^+^Th17 [helper T17] and CD8^+^Tc17 [cytotoxic T17] cells), innate-like lymphoid cells type 3 (ILC3), and gamma/delta [γ/δ] T cells which drive the initiation and propagation of immune responses preceding the development of chronic inflammation.

Axial spondyloarthritis (AxSpA) is characterized by spondylitis with the principal site of inflammation located at the enthesis leading to bone marrow oedema, infiltration of leuko, and lymphocytic cells, and increased osteoclast density. Cells and mediators of the innate immune system have a major role characterized by aberrant activity of dendritic, macrophage cells and innate-like immune cells, including γδ T cells, ILC3, neutrophils and mast cells. Development of chronic inflammation depends on production of pro-inflammatory lipids (including PGE2) produced by increased cyclo-oxygenase 2 (COX-2) expression/activity, which stimulates IL-17A production by Th17 cells, ILC3 and γ/δ T cells which is independent of IL-23 control.

Systemic Sclerosis (SSc) is characterized by extracellular matrix deposition and uncontrolled fibrogenesis whose development is related to the interplay of three major pathophysiological mechanisms, vascular impairment, autoimmunity and fibroblast dysfunction. Loss in T cell homeostasis leads to a reduction in Treg cells, enhanced autoreative CD4^+^T cells and B-cell production of autoantibodies, which play a central role in early disease. Activation of innate immune cells NK/NKT, pDCs, macrophage and mast cells contribute further to pro-inflammatory cytokine and growth factor secretion [IL-4, IL-6, TGFβ]. IL-4 can directly induce fibroblasts to promote fibrosis, but in addition, IL-4 induces TGFβ production in fibroblast and macrophage cells, leading to further signaling to fibroblasts and increased collagen production.

Osteoarthritis (OA) is characterized by less pronounced synovial tissue inflammation, but sufficient evidence supports its pathogenic role in disease progression and joint deterioration. Within OA synovium, inflammatory signatures including cellular hyper-proliferation, leukocyte and lymphocyte aggregate appearance, contribute to increased production of inflammatory molecules including TNF, IL-Iβ, Nitric Oxide (NO) and PGE2. The degree of inflammation is highly heterogeneous between patients, which is further supported by the lack of treatment responses and efficacy with anti-inflammatory treatments.

## Pivotal signalling pathways and targets of therapeutic strategies in rheumatic diseases

The identification of the cellular and soluble mediators driving the pathogenesis of IMRDs is contributing to the development of novel and emerging therapeutic strategies (8, 13, 14). Cellular components secrete specific cytokine signatures, and these mediators signal in an autocrine and/or paracrine manner to further activate intracellular pathways leading to altered transcription factor (TF) activity including IRAKs, RIPKs, JAK/STAT, AP-1, and NF-κB, that dictate cell-specific responses in IMRDs (15). Therapeutic approaches have been developed as a consequence of identifying these mediators and elucidating their signaling cascades in IMRDs, for example, the use of DMARDs, glucocorticoids, NSAIDs, small molecules (for example JAK/STAT, Rho GTPase inhibitors) and several biologics (bDMARDS) targeting various pathogenic factors involved (16); including, TNF, IL-6, and CD membrane proteins important for B- and T-cell function (CD20, CD80, and CD86, respectively) and co-stimulation blockers (13, 17). However, these therapies have high clearance rates, low bioavailability, and can be associated with systemic adverse effects, including but not limited to: gastrointestinal problems and increased risk of osteoporosis, infection and drug intolerance. Moreover, the response rate to individual therapies, such as TNF-based biologics, is variable and even after initial success relapse can occur for some patients, highlighting the heterogeneity of IMRDs (1). Regarding OA specifically, treatments have been limited to the use of surgery and analgesics to reduce pain (18). While TNF plays a role in OA pathology, recent clinical trials have shown the use of TNF based biologics are not efficacious as a treatment strategy (19, 20). At present, there is no DMOADs available for the treatment of OA, and treatment strategies are constrained (21). The adverse effects, limited therapeutic options for OA particularly, and the variable efficacy has reduced the overall success of current strategies, and it is evident that there is a need for more effective targeted therapeutics within the treatment of rheumatic diseases (22).

The JAK/STAT and the AP-1 pathways have also been shown to play important roles in arthritic disease, and similar to NF-κB display dysfunctional activity in early and late disease stages (23, 24). Additionally, these pathways have been shown to play specific roles in several innate and immune cell types in IMRDs, such as dendritic, macrophage and resident FLS cells (25–29). Novel JAK/STAT inhibitors have been recently approved in the treatment of RA with demonstrated significance in ameliorating immune-mediated inflammatory disease (30). Toll-like receptor (TLR) signaling and NF-κB activation has been long known to play a pivotal role in disease progression in IMRDs and OA (31). NF-κB plays comprehensive and diverse roles in arthritic disease, from regulating cytokine and chemokine production to cell specific regulation of multiple innate and adaptive immune cells, including dendritic, monocyte/macrophage, FLS, B- and T-cells (32, 33). NF-κB regulates the expression of numerous pathogenic mediators in inflammatory joint diseases such as TNF, IL-6, MMPs, MCP-1, growth factors and inducible COX-2 (leading to PGE2 production) (32). Nevertheless, the appropriate modulation of NF-κB activity has proven a difficult task, and as yet, NF-κB is an underdeveloped direct therapeutic target for diseases characterized with chronic inflammation (34). Importantly, the NR4A1-3 orphan receptors are established as prominent regulators of NF-κB signaling and activity in distinct cell types. NR4A1-3 receptors modulate NF-κB activity in a dynamic fashion, either repressing or enhancing target gene expression leading to altered inflammatory outcome and promoting inflammatory resolution (reviewed in 39 and 40). While the utility of the NR4A receptors as direct therapeutic targets is comparable to that of NF-κB, and to date hindered due to their difficulty in being directly druggable, this limitation has been overcome in recent studies (35–37). NR4A1-3 receptor activity and their cell specific functions in rheumatic disease are further delineated below.

## NR4A1-3 nuclear receptor activity and cell specific functions in rheumatic disease

The NR4A receptors are comprised of three family members: NR4A1 (Nur77), NR4A2 (Nurr1) and NR4A3 (Nor-1). The NR4As are one subfamily of the 48 members of the nuclear receptor (NR) superfamily (38–40). This NR superfamily includes transcription factors activated by ligand-binding and other members called ‘orphan' receptors, which have no known endogenous ligand. Endogenous ligands such as steroids, retinoids, and fatty acids bind NRs, and some well-established ligand controlled NRs include the glucocorticoid receptor (GR) and the peroxisome proliferator-activated receptor gamma (PPAR-γ) receptor (41–44). Members of the orphan class are constitutively active with their activity modified by several mechanisms, for example transcriptional regulation, post-translational modifications, co-factor binding and cellular localization (45, 46). At present the NR4A receptor subfamily reside in the ‘orphan' class, with their ligand-binding domain (LBD) established to be inhospitable to ligands, due to containing bulky amino acid side-chains (47). Significantly, recent studies establish that the LBD of NR4A2 is dynamic, with conformational changes occurring in micro-milliseconds, which in turn allows for the binding of unsaturated fatty acids (48, 49). The NR4A receptors, similar other NRs, contain an amino terminal domain (N-term), a DNA-binding domain (DBD) and a ligand binding domain (LBD), which display varying degrees of similarity between domains of all three NR4As, 26–28% (N-term), 94–95% (DBD), and 58–65% (LBD) (50). Given the similarities in all three NR4As LBD, and the likelihood of subfamily members sharing binding partners, their belonging to the ‘orphan' class may warrant some discussion (35, 36, 47).

The NR4A receptors are immediate early genes expressed in multiple cell types, in both early and late stages of diseases characterized by acute and chronic inflammation (38, 39, 51, 52). In IMRDs, NR4A subfamily members are aberrantly expressed in inflamed RA and PsA synovium, Ps dermal tissue, SSc fibrotic tissue and OA cartilage compared with normal tissue (53–57) NR4A1-3 receptor levels are induced by, and subsequently modulate cellular responses to key mediators in IMRD and OA, including TNF, IL-6, IL-1β, prostaglandins, growth factors and adenosine (58–60). Significantly, it is well-established that the NR4A receptors are critical modulators of TLR signaling and NF-κB regulated target genes in several cell types (38). There is an established role for sustained TLR activation in IMRDs, supporting a broad array of pathways activated in IMRDs that are regulated and counter-regulated by NR4A1-3 effector functions (52, 61, 62).

**Figure 1:**
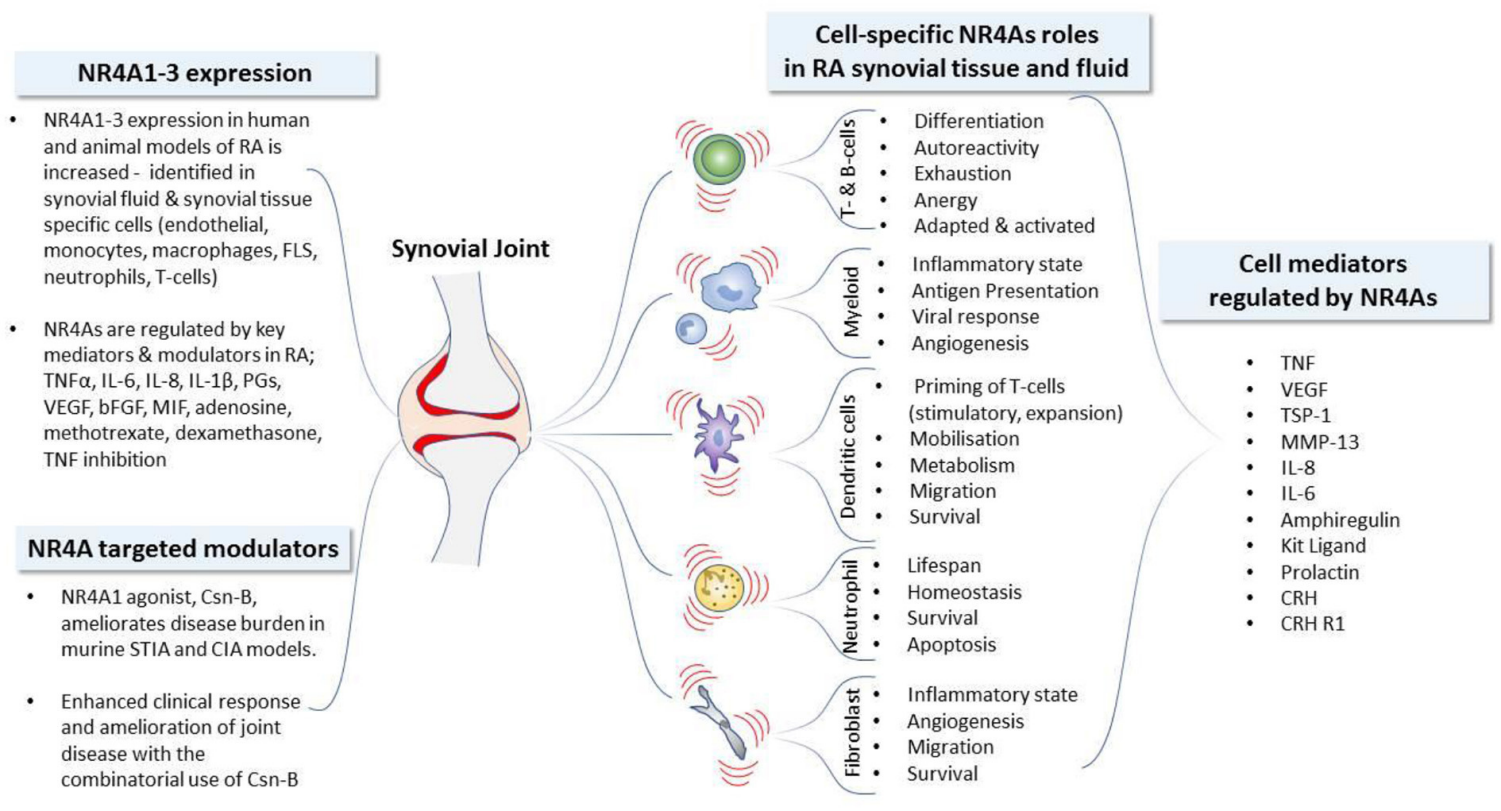
NR4A nuclear receptor activity and cell-specific functions in Rheumatoid Arthritis. Development and progression of immune mediated rheumatic diseases including rheumatoid arthritis (RA) are characterized by dysfunction of resident stromal fibroblast-like synoviocytes (FLS), innate and adaptive immune cell populations. The loss in cellular and tissue homeostasis culminates in a pro-inflammatory microenvironment leading to the advancement of chronic inflammation and disease progression. The expression profile of NR4A receptors identified in human and animal models of RA, together with the established mediators known to regulate *NR4A* receptor expression, are depicted. The functional activities of NR4As in synovial tissue and fluid is similarly cell-type dependent: NR4As regulate the synthesis and release of mediators to control angiogenesis, modulate the regulatory and invasive functions of resident FLS, alter the activation and tolerogenic status of dendritic cells, control homeostasis and the lifespan of neutrophils, influence the generation and responses of peripheral myeloid, B- and T-lymphocyte lineages and promote the maintenance of functional regulatory T-cells. The efficacious use of the NR4A1 modulator, Csn-B, reveals the therapeutic potential of NR4A receptors in models of RA with significant amelioration of disease activity. bFGF, basic fibroblast growth factor; CIA, collagen induced arthritis; CRH, corticotropin releasing hormone; CRH-R1, corticotropin releasing hormone receptor 1; Csn-B, cytosporone-B; FLS, fibroblast-like synoviocytes; KL, Kit ligand (also called SCF, stem cell factor); MIF, macrophage migration inhibitory factor; MMP, matrix metalloproteinase; PG, prostaglandin; IL, interleukin; STIA, serum transfer-induced arthritis; TSP-1, thrombospondin-1; TNF, tumor necrosis factor; VEGF, vascular endothelial growth factor.

Development and progression of IMRDs are characterized by dysfunction of resident endothelial, stromal fibroblast cells, innate and adaptive immune cell populations. The loss in cellular and tissue homeostasis culminates in a pro-inflammatory microenvironment leading to dysregulated repair, the advancement of chronic inflammation, disease progression and loss of tissue function. Target genes and physiological functions of NR4A1-3 receptors are tissue, cell type and context dependent (39, 40, 63). The cell- and tissue-specific events regulated by NR4A1-3 receptor activity and altered by NR4A1-3 receptor modulation in IMRDs are reviewed below. NR4A1-3 nuclear receptor regulation, activity, modulation and cell-specific functions in RA is summarized in Figure 1. NR4A1-3 involvement in cell-specific functional activities during rheumatic disease progression is further delineated below.

### Endothelial and fibroblast cells

Differential expression pattern of NR4A1-3 expression has been confirmed in primary endothelial and FLS cell populations isolated from inflamed human and mouse joint tissues suggesting that, within synovial tissue, the NR4A2 receptor may have a distinct role in regulating gene expression in response to inflammation (54, 64). The aberrant *in vivo* expression of NR4A2 in cells of the synovial lining layer and vascular endothelium confirms that NR4A2 is expressed in cells at the leading edge of invading pannus into cartilage. Enhanced NR4A2 activity induces a phenotypic shift in normal FLSs that parallels the cellular transformation and hyperplasia observed during the progression of IMRD (57). Through the generation of stable and transient NR4A2-expressing FLSs, *in vitro* cell systems, reflecting the characteristics of activated primary FLS, were achieved. Using such cell systems, it was established that NR4A2-overexpressing clones and low-passage primary RA FLS express high VEGF-A, MMP-13, IL-8, amphiregulin and prolactin mRNA and protein levels (57, 65–67). Furthermore, the anti-angiogenic matrix protein, thrompospondin-1 (TSP-1) mRNA and secreted protein levels were significantly reduced. Modulation of TSP-1 expression in RA synovial tissue can be achieved *in vivo* through TNFi therapy effects on transcriptional networks including NR4A2 activity, suggesting that altered TSP-1 levels early in disease may be beneficial, leading to inflammatory resolution (56). These results identify TSP-1 as a target of NR4A2 activity and are consistent with studies proposing a pro-angiogenic role for the NR4A subfamily *in vivo* models of disease (68, 69). Angiogenesis and microvessel permeability induced by VEGF-A, histamine or serotonin are almost completely inhibited in NR4A1 knockout mice (70). Furthermore, NR4A1 functions as a critical mediator of pathological angiogenesis (71–74), and established as a feasible target for pro-angiogenic and anti-angiogenic therapies with translational potential (71).

NR4A1 expression is significantly elevated in fibroblast cells isolated from fibrotic conditions (75, 76) including fibrotic skin of patients with SSc (57). A study by Pulumbo-Zerr et al. (57) demonstrates that NR4A1 acts a regulator of normal wound repair responses with exacerbation of fibrotic disease in several models of cutaneous fibrosis in NR4A1^−/−^ mice. The regulatory effects of NR4A1 are lost in fibrotic disease leading to hyper TGFβ signaling and pro-fibrotic effects. During fibrosis, treatment with the NR4A1 agonist, Cytosporone B (CsnB), can reactivate NR4A1, leading to reduced TGFβ signaling, fibrosis inhibition and restoration of normal repair responses (57).

### Monocyte and macrophage cells

Consistent with findings that NR4A2 is expressed by macrophage cells in RA ST and in several models of murine autoimmune joint disease, treatment of monocyte and macrophage cells with diverse mediators including lipopolysaccharide (LPS), cytokines, growth factors or oxidized lipids triggers the transcriptional induction of all three NR4A (38, 54). It is recognized that NR4A1-3 stimulus- and cell context-dependent activities, controlling pathological processes (through genomic and non-genomic actions), are influenced by their relative expression levels, subcellular localization, interactions with co-factor proteins and changes in cellular metabolism (39, 63, 77).

NR4A1 is required in the differentiation and survival of Ly6C- monocytes and for the development and maturation of infiltrating Ly6C+ and Ly6C- monocytes from myeloid dendritic precursors (78, 79). In a dermal fibrosis model of SSc, treatment with a NR4A1 agonist, CsnB, leads to conversion of pro-inflammatory monocytes Ly6C+ monocytes to patrolling Ly6C- monocytes with NR4A1-dependent resolution of cutaneous fibrosis (80). The role of NR4A1 depletion in the development of macrophage polarization toward a pro-inflammatory M1 phenotype with enhanced TLR signaling is further documented (81). In a serum transfer-induced arthritis (STIA) model NR4A1-dependent Ly6C- monocytes contribute to reducing joint inflammation in arthritic mice through the mobilization of Treg cells (82). Recent transcriptional profiling of monocytes deficient in NR4A2 and NR4A3 reveals distinct signaling roles in mediating antigen presentation and responses to viral infection, respectively (83).

### Dendritic cells

Dendritic cells (DC) are the most specialized and efficient in the priming of de novo T-cell responses and thus serve as a critical bridge between innate and adaptive immunity. Elucidating the immuno-modulatory effects of NR4A1-3 during DC differentiation and DC function has been a focus of several recent studies (84–86). NR4A1-deficient DCs show enhanced inflammatory responses, leading to enhanced NF-κB dependent cytokine production and T-cell stimulatory capacity of DCs (84). Exogenous NR4A2 has been shown to restrict the immunogenicity of bone marrow derived DCs (BMDCs) activated by LPS (87). The adoptive transfer of BMDCs overexpressing NR4A2 in mice induced expansion of Treg cells and restricted the abundance of T effector cells *in vivo* suggesting that NR4A2 is a critical regulator of DC tolerogenicity (tolDCs). NR4A3 leads to activation-induced cell death in DCs, is critically important in CD107^+^ DC migration through TLR-dependent and -independent regulation of CCR7, is further involved in mitochondrial function during DC maturation and TLR-mediated activation/gene expression of DCs (85, 88).

### T and B lymphocyte cells

NR4A family members are highly homologous proteins and can have redundant functions as seen in their functional roles to establish and maintain regulatory T-cell identity (89). FOX3P^+^ Treg development is complemented by NR4A family members, and only through combined deletion of NR4A1, NR4A2, and NR4A3 is FOX3P^+^ T cell development ablated *in vivo* (90). NR4A1-3 receptors are upregulated in T and B cells after acute antigen encounter, in Tregs in the steady state, in thymocytes undergoing negative selection, and in self-reactive, anergic, or exhausted lymphocytes in response to chronic antigen stimulation (recently reviewed in 44). Indeed, the NR4A family has been argued to play central and peripheral tolerogenic roles in all these contexts (91).

NR4A1 and NR4A3 also play non-redundant roles in peripheral conventional T-cells: most notable among these are roles for NR4A1 in CD4^+^ Th cell anergy and an additive role for all three NR4A family members in CD8^+^ Th cell exhaustion (92). Gene expression data show that endogenous Nr4a1 transcripts are highly up-regulated in autoantigen-specific CD4^+^Th cells *in vivo* in the context of autoimmune disease (93, 94). Such profiling indicates that NR4A1 expression could be employed to identify autoantigen-specific CD4^+^Th cells in RA. Consequently endogenous NR4A1 has been utilized as a marker of T cell antigen receptor (TCR) signaling-to identify antigen-activated CD4^+^ Th cells in the SKG mouse model of autoimmune arthritis and in patients with RA. Using a fluorescent reporter of NR4A1 expression in SKG mice, it was established that higher levels of NR4A1-eGFP in SKG CD4^+^Th cells marked their auto-reactivity, arthritogenic potential, and ability to more readily differentiate into Th17 cells with greatly enhanced IL-6-dependent signaling. In addition, all three *NR4A* receptors display differential CD4^+^ Th cell expression at onset and at the fully developed state of disease in the collagen-induced arthritis (CIA) model of RA disease (95).

Integrated single cell analysis of RA synovial tissue B lymphocyte cells identify a unique activated B-cell population characterized by NR4A receptor expression. NR4A^+^ B-cells express ectopic lymphoid structure (ELS) chemotactic factors (LT-α, LT-β and IL-6) and demonstrate loss of a naive B-cell state. Collectively, these data suggest NR4A receptors have an important role in local adaptive B-cell responses with NR4A expression acting as a potent readout of pathogenic B-cells in RA (96).

### Neutrophil cells

Dysregulated neutrophil activation contributes to the pathogenesis of IMRDs influencing the phenotype and function of other innate and adaptive immune cells (97). Spatial and temporal control of neutrophil turnover is critical for resolution of inflammation, and alterations in this process exemplify an aberrant and dysregulated inflammatory response that leads to tissue destruction and loss of function. Accordingly, as part of the Immunological Genome Project (93, 94), gene expression was determined in unstimulated (circulating) mouse neutrophils and three populations of neutrophils activated *in vivo*, with comparison of gene expression patterns among these populations (98). Activation conditions included serum-transfer induced arthritis (STIA) (mediated by immune complexes), thioglycollate-induced peritonitis (TG), and uric acid-induced peritonitis (UA). *Nr4a2* and *Nr4a3* expression is up-regulated only in synovial fluid neutrophils elicited by STIA arthritis, and *Nr4a1* expression is up-regulated in STIA and to a lesser extent in TG neutrophils. *Nr4a1-3* gene expression remained unchanged in UA neutrophils. Further compelling evidence indicates that NR4A2 and NR4A3 are critical regulators of neutrophil homeostasis and lifespan controlled via a cAMP-PKA-NR4A2/3-neutrophil survival signaling pathways (99, 100). Using ultrapure human neutrophils in gene array studies, PKA activation upregulates a significant number of apoptosis-related genes, the most highly regulated include NR4A2 and NR4A3. Direct PKA activation and treatment with endogenous activators of PKA, including adenosine and PGE2, results in a profound delay of neutrophil apoptosis and concomitant upregulation of NR4A2/3 in a PKA-dependent manner (99).

### Cartilage

Expression of the NR4A1-3 receptors in human OA cartilage is significantly higher than levels measured in normal cartilage (101, 102). Consistent with advanced cartilage degradation and inflammation, cyclooxygenase-2 (COX-2) levels are also significantly elevated and correlate significantly with changes in NR4A2 gene expression (100). Interestingly, COX-2-derived PGE_2_ rapidly and potently induces NR4A2 expression in primary human chondrocytes. Protective functions for NR4A1 and NR4A2 in human cartilage homeostasis by selectively repressing MMP-1,-3 and−9 gene expression during inflammation have been established (101). In a rat model of OA, NR4A1 is a key endogenous inhibitor of chondrocyte inflammation. This OA study shows that, during chronic inflammation, NR4A1 is inactivated through HDACs mediated transcriptional suppression and MAKP dependent phosphorylation. Intra-articular (IA) administration of the NR4A1 agonist, Csn-B, reactivates and restores the anti-inflammatory effects of NR4A1, preventing excessive inflammation [through the repression of IL-1β-induced chondrocyte inflammation and expression of COX-2, iNOS, MMP-3, MMP-9, and MMP-13] and ameliorating cartilage degradation and osteoarthritis progression (103). In contrast, NR4A3 may mediate a pro-inflammatory role in mediating cartilage homeostasis and OA pathogenesis (104); collectively these studies demonstrate both pro- and anti-inflammatory functions for NR4A1-3, proposing that acute or chronic levels of inflammation permit differential effects on NR4A receptor expression and activity.

## NR4A1-3 receptors and emerging therapeutic strategies in rheumatic diseases

Modulation of NR4A receptor activity can influence disease outcome, as demonstrated in acute myeloid leukemia (AML), with the identification of specific drugs that modulate NR4A1/3 expression and NR4A-gene signatures in AML cells (105, 106). Current reports propose that therapeutic approaches targeting tissue resident synovial cells in RA could potentially restore homeostasis (107). Recently, the use of Csn-B, an NR4A1 ligand, has been shown to ameliorate disease burden in a murine serum transfer-induced arthritis (STIA) model, in a collagen-induced (CIA) model and in a rat model of OA (82, 95, 103). In the CIA model, the single or combinatorial use of Csn-B, with two further synthetic ligands, CD437 and CGP 52608, was used to selectively target NR4A1, NR0B2, and RORa, respectively. Significantly, the combined treatment of these synthetic ligands was found to be more effective than when treated with individual ligands; with combined-treated animals displaying superior protection resulting in resolution to an almost normal synovium (95).

Moreover, within the in *vivo* models described (95), Csn-B was delivered through intraperitoneal or IA injection; it was not cell-type specific. Considering that these orphan receptors mediate cytokine, prostanoid and growth factor signaling to control cellular and secreted components of resident, innate and adaptive immune cell responses, targeting NR4A1-3 receptors in a cell-type directed approach to control inflammatory disease outcome may be a viable option in IMRDs (39, 52, 108). Integrating knowledge of NR4A1-3 modulation and the impact on cell- and tissue-specific functional activities, at distinct stages during chronic inflammation, will facilitate the development of new therapeutic targets and strategies. To complement progression in the potential application of NR4A ligands as viable therapeutic options are recent developments in drug delivery systems (109). For example in the field of nanomedicine, the development of precision nanocarriers, which can be modified to enable site- and cell-specific delivery, have been loaded with therapeutic agents, including steroids, are showing improved permeability and retention in the joint space, with enhanced bioavailability and bioactivity, and reduced clearance rates (110, 111). Furthermore, such advances can augment drug efficacy and reduce unwanted systemic adverse effects (109).

Importantly, further recent developments in cell-based therapeutic advances in the field include IA delivery of mesenchymal stem cells (MSCs) (112–114). MSCs can evade immune surveillance, thereby escaping rejection, and possess immunomodulatory abilities rendering them an excellent therapeutic candidate. Another practical benefit is that they are relatively simple to isolate and expand *in vitro* (114). Multiple clinical trials using MSCs in immune related disorders are proceeding, including RA and OA (115, 116). While the trials are showing therapeutic and clinical promise, the exact longevity of their retention in the joint space is unclear. Another therapeutic option for RA and OA is that of IA delivery of tolerogenic dendritic cells (tolDCs), which have the capacity to restore immune homeostasis (117, 118). Regarding tolDCs, phase I trials have already shown that they are safe and feasible, without adverse effects being observed (119, 120). As underscored, acquiring comprehensive knowledge regarding the contribution of cell-specific activities in disease initiation, progression and resolution, together with the development of targeted, site- and cell-specific delivery systems, will undoubtedly provide a useful arm of future therapeutic approaches, of both ‘old' (for example steroids) and ‘new' (for example NR4A modulators) drugs (121, 122).

## Conclusion

Our understanding of the pathogenesis of IMRDs has advanced to a degree that allows each disease to be stratified based on their shared and individual properties. Such disease features include, but are not limited to; cell types of importance, intracellular regulatory processes, transcription factor activity, secreted mediators and responses to therapies. In this review, we describe how NR4A1-3 orphan nuclear receptors, whose expression and activity is altered in several IMRDs, are key regulators in multiple cell types within these diseases, including endothelial, fibroblasts, neutrophil, dendritic, monocytes, macrophage, T-and B-lymphocyte cells. Mechanistically, the NR4A receptors affect these cell types across a broad spectrum; regulating signaling pathways, activation status, cell-cell interactions, migration capacity, metabolism, and survival. Moreover, these cellular alterations to which the NR4As are intrinsic, can lead to tissue changes in early and late stages of disease progression and during inflammatory resolution. At present, key therapeutic advances in the treatment for IMRDS, including the development of MSCs and tolDCs are developing. Given the central functions NR4A1-3 receptors control, we propose that these nuclear receptors may perform as feasible adjunct targets in the development of cell-specific therapies in immune mediated rheumatic diseases.

## Author contributions

All authors listed have made a substantial, direct, and intellectual contribution to the work and approved it for publication.

## Funding

This work was funded by the School of Medicine, University of Limerick, Ireland.

## Conflict of interest

The authors declare that the research was conducted in the absence of any commercial or financial relationships that could be construed as a potential conflict of interest.

## Publisher's note

All claims expressed in this article are solely those of the authors and do not necessarily represent those of their affiliated organizations, or those of the publisher, the editors and the reviewers. Any product that may be evaluated in this article, or claim that may be made by its manufacturer, is not guaranteed or endorsed by the publisher.
